# Adherence to treatment in collective multiprofessional activities and factors associated with adherence in a specialized center for psychosocial care

**DOI:** 10.1590/2237-6089-2019-0053

**Published:** 2020-08-14

**Authors:** Letícia P. Tergolina, Airton T. Stein, Evelise R. de Faria

**Affiliations:** 1 Grupo Hospitalar Conceição Porto AlegreRS Brazil Grupo Hospitalar Conceição (GHC), Porto Alegre , RS , Brazil .; 2 Universidade Federal de Ciências da Saúde de Porto Alegre Porto AlegreRS Brazil Universidade Federal de Ciências da Saúde de Porto Alegre (UFCSPA), Porto Alegre , RS , Brazil .

**Keywords:** Mental health services, mental disorders, rehabilitation, socialization, patient care team, patient compliance

## Abstract

**Introduction:**

Specialized psychosocial care centers (Centros de Atenção Psicossocial [CAPS]) are mental health services focused on social rehabilitation and reducing hospitalization of patients with severe and persistent mental illness. Collective multiprofessional activities (CMPA) are the main therapeutic tools used at CAPS. This study aimed to determine rates of adherence to CMPA and identify factors associated with adherence.

**Methods:**

This is a cross-sectional study in which 111 CAPS users were evaluated using questionnaires covering patient characteristics, clinical status, and treatment and incorporating the Functioning Assessment Short Test (FAST), the Clinical Global Impression – Severity scale (CGI-S), and the Clinical Global Impression – Improvement scale (CGI-I). Adherence was defined as attendance at 50% or more CMPA during the previous 3 months. Data were analyzed using descriptive statistics, bivariate analysis, and Poisson logistic regression with robust variance to estimate prevalence ratios.

**Results:**

CPMA adherence was 43%. Having children aged 14 years or younger was significantly associated with non-adherence (71%, p = 0.001). Poor or partial adherence to psychotropic drugs tended to be associated (p = 0.066) with poor adherence (33% higher risk), as was the number of psychiatric hospitalizations during CAPS (p = 0.076), with a cumulative association of 5% non-adherence per hospitalization.

**Conclusions:**

CMPA adherence was low in the study. It is necessary to consider the environment in which the individual lives and invest in support networks, providing patients and family members with explanations about the importance of CMPA to rehabilitation and attempting to tailor the care provided to each patient’s needs. There was an association between greater number of psychiatric hospitalizations and non-adherence, suggesting that CAPS are fulfilling a preventive role.

## Introduction

Specialized psychosocial care centers (Centro de Atenção Psicossocial [CAPS]) are regionalized mental health centers providing secondary care, with the main objective of promoting psychosocial rehabilitation and reducing psychiatric hospitalizations and institutionalizations. They have interdisciplinary teams and are known for their therapeutic groups and workshops, here referred to as collective multiprofessional activities (CMPA). ^1 - 3^ The expected profile of patients seen at CAPS is those with severe and persistent mental illness (SPMI), ^2 , 3^ represented by psychotic disorders. ^4^ A previous study, conducted in northeastern Brazil, found a 64% prevalence of psychotic disorders among CAPS patients and a 36% prevalence of mood disorders. ^5^ SPMI are characterized by chronicity and clinical severity and by incapacity to perform social functions, with impaired autonomy, poor social support, isolation, and poor prognosis. ^6 , 7^ These patients tend to lack financial independence, housing, and work and have poor social interaction, motivation, and cognition, with high social costs. ^4^

A previous literature review found limited response to pharmacological treatment in terms of social functioning improvements in patients with SPMI. ^8^ Psychosocial rehabilitation has been the approach of choice in these cases and is a process of achieving the best level of autonomy possible for exercise of social functions. ^9 - 11^ Psychosocial rehabilitation involves access to work, leisure, exercise of civil rights, and strengthening of family and community ties. ^12 , 13^ The main psychosocial rehabilitation tool used at the CAPS are CMPA, which provide opportunities for systematic social experimentation in a safe therapeutic space. ^4 , 14^ CMPA favor understanding, support, acceptance, and exchanges between patients, reducing isolation and offering a restorative social experience in order to develop new ways of relating. ^14 - 16^ Literature suggests that patients who regularly attend psychosocial rehabilitation services feel more satisfied with their relationships and less alone. ^17^ Despite this potential, clinical experience suggests that a large proportion of patients have low adherence to CMPA, compared to consultations and use of psychotropic drugs.

There is no clear definition of the concept of group treatment adherence in the literature. ^18^ A study focused on treatment adherence in the Brazilian mental health context ^18^ did not identify studies of adherence to psychosocial rehabilitation treatment using CMPA, since the literature refers almost exclusively to adherence to medication. There has been minimal investigation of the complexity of the interdisciplinary treatment offered by CAPS, which is geared toward individualized therapeutic rehabilitation. ^18^ Another study analyzed scientific literature on CAPS, identifying 68 references. ^13^ Most of them were qualitative (95.5%) and only two (3%) quantitative studies were identified. With regard to the authors of these studies, 50% were nurses, 16.5% were psychologists, and 8.5% were physicians. The main topics addressed were mental health policy, professionals’, patients’, and family members’ perspectives of the service, and analysis of CAPS practices. Only two studies evaluated the effectiveness, and user profiles (3%) of therapeutic groups. No studies on CMPA adherence were identified.

Although CMPA is the main tool used for rehabilitation of patients at CAPS, no studies were found that assess their adherence level and factors associated with adherence. Prior to starting this study, a hypothesis was raised that patients with more stable clinical status, better psychosocial functioning, more schooling, higher income, free transportation, and better support networks would have better adherence. The aims of the study were to evaluate the CMPA adherence levels of patients undergoing active treatment at a CAPS and to identify factors associated with adherence, considering patient characteristics, treatment context, and service characteristics.

## Method

A cross-sectional study was conducted to measure level of adherence to CMPA and factors associated with adherence at a CAPS located in Porto Alegre, Brazil. The service is classified as a “CAPS 2” and serves a population of 270,000 inhabitants, which is a larger population than would be expected for the CAPS 2 classification (a population of 70,000 to 200,000 inhabitants ^1^ ). The CAPS 2 is open Monday to Friday from 8am to 6pm. Its clinical staff include psychiatrists, psychologists, a social worker, an occupational therapist, an art therapist, nurses, and nursing technicians. The service also hosts interns and residents on placements from healthcare areas. The CMPA provided at this CAPS include approximately a dozen workshops and therapeutic groups held on a weekly basis. Each CMPA is conducted by one (sometimes two) of the staff members, usually with the participation of the temporary students.

Data were collected between December 2016 and May 2017. At that time, a total of 189 patients were being cared for at the CAPS. The inclusion criteria were: 1) actively treated at the CAPS with regular attendance at psychiatric consultations (defined as 50% or greater attendance at scheduled appointments); 2) scheduled for participation in CMPA; 3) attending the service for at least 4 months. Exclusion criteria were: 1) a primary diagnosis of mental retardation, autism, or other neurological disease; and 2) inability to respond to the questionnaire due to symptom severity. All the information for both sets of criteria was obtained from the professionals that care for the patients and later checked against the medical records.

Patients were invited to participate in the research on the days they were at the CAPS for their usual therapeutic activities. Those who agreed to participate received and signed two copies of the Free and Informed Consent Agreement, keeping one of them. After agreement, information was collected from medical records and then the interview was conducted.

Data were collected by two interviewers who were CAPS staff members. One was the psychiatrist who designed this study and the other was a nurse who was given a two-hour training session (by the first researcher) on the study objectives and instruments. Interviews were conducted individually in the CAPS meeting rooms and lasted approximately 20 minutes.

Data collection involved administration of a questionnaire covering patient characteristics, clinical status, and current and previous treatment, consisting of 77 items and incorporating three validated scales. Medical records were also reviewed and additional information was collected from the center’s service professionals.

The study outcome – level of adherence to CMPA over the previous three months – was calculated using attendance lists and medical records from the three months preceding the interview. Attendance was considered based on CMPA sessions that actually took place, disregarding holidays observed by the service or by therapists leading group. Participants who had a 50% or greater rate of attendance at these activities were defined as adherent according to a clinical definition. This was considered the minimum level of activity involvement required to achieve therapeutic gains. All participants had monthly appointments with their psychiatrist as part of the standard CAPS routine, regardless of their CMPA adherence.

Participants also answered an open-ended question about their reasons for absenteeism from the collective activities. Subsequently, their reasons were grouped into the following categories: 1) Motivational; 2) Financial; 3) Psychiatric disease; 4) Clinical Illness/Medical Commitments; 5) Other Commitments/Family Cares; 6) Work.

Regarding independent variables, the following sociodemographic and economic data were collected from patients’ answers to the questionnaire: age, gender, ethnicity, marital status, number of children and their ages, schooling, occupation, years of employment, years unemployed, per capita family income, free transportation. Social support levels were assessed based on the patient’s account of who were the people that provided them with support and how much they felt supported by them. Psychiatric diagnoses were obtained from the medical records according to the CAPS treating psychiatrist’s impressions and later classified as Psychotic (Schizophrenia or Schizoaffective Disorder) or Non Psychotic (Bipolar Disorder, Depression Disorder, Anxiety Disorders, Stress Disorders, or Obsessive-Compulsive Disorder). Presence of a personality disorder was also identified in the medical records according to the treating psychiatrist’s evaluation. Other variables related to illness were: time since diagnosis/in treatment, clinical multi-morbidities, smoking, and suicide attempts, all collected by directly asking patients for information. The questionnaire also addressed pre-CAPS mental health treatment, including previous psychiatric hospitalizations, as well as information about the quality of the therapeutic bond with the basic health unit, psychiatric hospitalizations after entry, whether they felt supported by CAPS, whether they valued collective activities, and whether they had social activities outside (work, gym, classes, groups, workshops, church), all obtained by patient report. Variables like time in treatment at CAPS, type of CMPA attended, and individual treatments at CAPS were accessed from medical records. Adherence to psychotropic drugs was evaluated by patient report. Poor or partial adherence was defined as when patients stated they were not taking some of their medications or were frequently forgetting to take them, while good adherence was defined as when they responded that they never or very rarely missed taking their drugs.

Three scales were administered: the Functioning Assessment Short Test (FAST), with an overall score ranging from 0 to 72, evaluates global psychosocial functioning and its domains: autonomy (0-12), occupation (0-15), cognitive functioning (0-15), financial management (0-6), interpersonal relationships (0-18), and leisure (0-6) ^19^ ; the Clinical Global Impression – Severity scale (CGI-S), scored from 1 to 7, evaluates current symptom severity, and the Clinical Global Impression – Improvement scale (CGI-I), also scored from 1 to 7, evaluates the degree of improvement or worsening in relation to the start of treatment. ^18^ All three scales have been validated for the Brazilian population. ^19 , 20^

Data were analyzed using SPSS software, version 22. Descriptive statistics were calculated (percentages for categorical variables, means and standard deviations for quantitative variables). Comparisons between categorical variables were conducted using the chi-square test. Comparisons between quantitative variables were conducted using Student’s t test.

Bivariate comparisons were made between the adherence group (50% or greater attendance at collective activities) and the non-adherence group. Variables that had p < 0.1 in the bivariate analysis were pre-selected to enter the multivariate analysis, also taking into account their clinical relevance.

This investigation was judged ethically and methodologically appropriate by the Research Ethics Committee at the Grupo Hospitalar Conceição (protocol number 16250) and conducted in line with the standards required by the Declaration of Helsinki.

## Results

**Figure 1:**
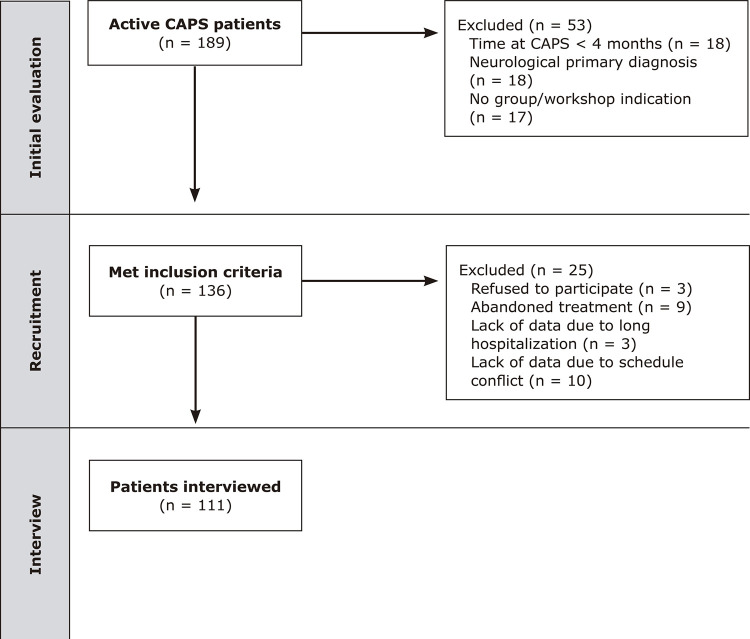
Study flowchart. CAPS = Centros de Atenção Psicossocial (psychosocial care centers).

At the beginning of data collection, 136 CAPS patients met the inclusion criteria for the study. Twenty-five of these people could not be included because they refused to participate or were unavailable for the interview, so the final sample comprised 111 patients ( Figure 1 ).

The main reason patients reported for not attending CMPA was lack of motivation (36%). Symptoms of their psychiatric disorders were reported by 28% (feeling very depressed, anxious, irritable, having difficulty coexisting with others, or going out alone). Other reasons given included: financial difficulties (19%), social commitments and care of relatives (18%), clinical diseases or medical commitments (16%), and work (8%).

Adherence to collective activities was 43%, even though 72% of participants evaluated these activities as of great importance for their treatment. In comparison, 67% reported good adherence to psychotropic drugs and all attended regular psychiatric consultations. Despite having been scheduled for CMPA as part of their therapy, 45% never attended during the 3 months assessed. Table 1 lists the participants’ characteristics.

**Table 1:** Description of the population – sociodemographic and clinical variables

Participants	111 (100)
Adherence	48 (43.2)
Has collective care	61 (54.9)
Age (years), mean (SD)	42.3 (10.7)
	
Gender, female	68 (61.3)
	
Ethnicity	
Caucasian	73 (65.8)
Afro-Brazilian/mixed race	38 (34.2)
	
Marital status	
No companion	63 (56.8)
	
Has children	71 (64)
Number of children, median (IQR)	1 (0;3)
Child aged 14 years or younger	29 (26.4)
	
Schooling (years), mean (SD)	8.7 (3.1)
	
Occupation	
Employed	10 (9)
Unemployed/unpaid	40 (36)
Sickness benefit	39 (35.1)
Permanent benefit	22 (19.8)
	
Time unemployed (years), median (IQR)	4 (4;8)
Family income per capita - BRL, median (IQR)	620.00 (366.66;1.000.00)
Receives free transportation	41 (36.9)
	
Social support	
Little	30 (27)
Significant	81 (73)
	
Psychotic diagnosis*	45 (40.5)
Non-psychotic diagnosis ^†^	66 (59.4)
Time (years) since diagnosis/in treatment, median (IQR)	10 (5;16)
	
Personality disorder	30 (27)
Borderline	10 (9)
Histrionic	13 (11.7)
Other	8 (7.2)
	
CGI-S, median (IQR)	4 (4;5)
CGI-I, median (IQR)	2 (2;3)
Previous psychiatric hospitalization	67 (60.4)
Psychiatric hospitalization during time at CAPS	33 (29.7)
Number of psychiatric hospitalizations during time at CAPS, median (IQR)	1 (0;3)
	
FAST scores, mean (SD)	
FAST Total (0-72)	48.3 (11.1)
FAST Autonomy (0-12)	6.3 (2.6)
FAST Work (0-15)	14.0 (3.0)
FAST Cognition (0-15)	9.7 (3.5)
FAST Finances (0-6)	3.8 (1.9)
FAST Interpersonal Relationships (0-18)	10.5 (3.9)
FAST Leisure (0-6)	3.9 (1.6)
	
Weeks in CAPS treatment, median (IQR)	24.5 (7;48)
	
Adherence to psychotropic drugs	
Poor/partial	36 (32.4)
Good	75 (67.6)
	
Importance of collective activities	80 (72.1)

Data presented as n (%), unless otherwise specified.

CAPS = Centros de Atenção Psicossocial (psychosocial care centers); CGI-S = Clinical Global Impression – Severity scale; CGI-I = Clinical Global Impression – Improvement scale; FAST = Functioning Assessment Short Test; IQR = interquartile range (25th;75th percentile); SD = standard deviation.

* Schizophrenia/schizoaffective disorder.

^†^ Bipolar disorder/depressive disorder/anxiety disorder/obsessive compulsive disorder/stress.

The main reason given for absences by patients who were noncompliant with collective activities was motivational (43%), followed by psychiatric symptoms (30%), and financial difficulties (22%). Participants with adequate adherence said that they were mainly absent due to clinical diseases and medical commitments (31%), followed by motivational factors (27.1%), other commitments/caring for family members (27.1%), and psychiatric symptoms (25%).

A bivariate analysis was conducted comparing the adherent group with the non-adherent group ( Table 2 ). There were no differences between the groups regarding severity and symptomatic improvement, or psychosocial functioning, except in the FAST Interpersonal Relations domain. The same was observed for gender, age, marital status, and time in treatment at the CAPS. Reports of low social support, personality disorder, psychiatric hospitalization during CAPS, recent work leave, employed or on sickness benefit, free transportation, and higher income were also not associated with better adherence to CMPA.

**Table 2:** Bivariate analysis

	Adherence (n = 48)	Non-adherence (n = 63)	p	PR	95%CI
Child ≤ 14 years old					
No	44 (54.3)	37 (45.7)			
Yes	4 (13.8)	25 (86.2)	**< 0.001**	**1.887**	**1.428-2.493**
					
Number of children, median (IQR)	1 (0;2)	2 (0;3)	**0.038**	**1.098**	**1.005-1.201**
					
Occupation					
Employed	2 (20.0)	8 (80.0)	-	1	-
Unemployed/unpaid	18 (45.0)	22 (55.0)	0.079	0.687	0.453-1.044
Sickness benefit	15 (38.5)	24 (61.5)	0.195	0.769	0.517-1.144
Permanent benefit	13 (59.1)	9 (40.9)	**0.026**	**0.511**	**0.283-0.923**
					
Free transportation benefit					
No	28 (40.0)	42 (60.0)	0.382	1.171	0.822-1.670
Yes	20 (48.8)	21 (51.2)			
					
Psychotic diagnosis*	24 (53.3)	21 (46.7)			
Non-psychotic diagnosis ^†^	24 (36.4)	42 (63.6)	0.093	1.364	0.950-1.958
					
Adherence to psychotropic drugs					
Poor/partial	11 (30.6)	25 (69.4)	**0.047**	**1.371**	**1.004-1.871**
Good	37 (49.3)	38 (50.7)			
					
Importance of collective activities					
Little	4 (12.9)	27 (87.1)	**< 0.001**	**1.935**	**1.466-2.555**
Significant	44 (55.0)	36 (45.0)			
					
Psychiatric hospitalizations during CAPS, median (IQR)	0 (0;0)	0 (0;1)	**0.022**	**1.055**	**1.008-1.104**
Time in years since diagnosis/in treatment, median (IQR)	10 (7;20)	8 (4;13)	0.064	0.980	0.959-1.001
					
FAST Interpersonal Relationships, mean (SD) ^‡^	11.1 (3.0)	10.0 (4.5)	0.077	0.970	0.938-1.003

Data presented as n (%), unless otherwise specified.

95%CI = 95% confidence interval; CAPS = Centros de Atenção Psicossocial (psychosocial care centers); FAST = Functioning Assessment Short Test; IQR = interquartile range (25th;75th percentile); PR = prevalence ratio (PR < 1 = adherence; PR > 1 = non-adherence); SD = standard deviation.

Bold type indicates statistically significant values (p < 0.05) .

* Schizophrenia/schizoaffective disorder

^†^ Bipolar disorder/depressive disorder/anxiety disorder/obsessive compulsive disorder/stress.

^‡^ Scores range from 0-18.

A higher number of children (p = 0.038) and having children aged 14 years or less (p < 0.001) were associated with poor adherence to CMPA. Regarding occupation, receiving permanent benefits (p = 0.026) was associated with better adherence. There was also a significant association between a greater number of psychiatric hospitalizations after admission to the CAPS and worse adherence (p = 0.022). Patients with poor or partial psychotropic drug adherence also came to the group meetings less frequently (p = 0.047). Those who stated that they did not consider collective activities important presented worse adherence (p < 0.001)

Some variables indicated a statistical trend to difference between the groups, that was not significant, but indicated better adherence among participants with longer time since onset of diagnosis and treatment (p = 0.064), psychotic underlying diagnosis (p = 0.093), and higher scores in the FAST Interpersonal Relations domain (p = 0.077). With regard to occupation, not having an income had a trend to association with worse adherence (p = 0.079).

A multivariate analysis was performed using Poisson logistic regression, with seven eligible variables in the model: having a child aged 14 years or younger, occupation, psychotic diagnosis, adherence to psychotropic drugs, number of psychiatric hospitalizations during time at CAPS, time of onset of diagnosis/treatment, and FAST Interpersonal Relationships score ( Table 3 ).

**Table 3:** Poisson logistic regression

	p	PR	95%CI
Child ≤ 14 years old			
No			
Yes	**0.001**	**1.70**	**1.243-2.349**
			
Occupation			
Employed			
Unemployed/unpaid	0.204	0.77	0.523-1.148
Sickness benefit	0.926	0.98	0.636-1.510
Permanent benefit	0.261	0.68	0.355-1.324
			
Psychotic diagnosis*			
Non-psychotic diagnosis ^†^	0.542	1.12	0.764-1.669
Adherence to psychotropic drugs			
Poor/partial	0.066	1.33	0.982-1.812
Good			
			
Number of psychiatric hospitalizations during CAPS	0.076	1.04	0.995-1.105
Diagnostic/treatment time – years	0.299	0.98	0.967-1.011
FAST Interpersonal Relationships (0-18)	0.485	0.98	0.955-1.022

95%CI = 95% confidence interval; CAPS = Centros de Atenção Psicossocial (psychosocial care centers); FAST = Functioning Assessment Short Test; PR = prevalence ratio (PR < 1 = adherence; PR > 1 = non-adherence).

Bold type indicates statistically significant values (p < 0.05).

* Schizophrenia/schizoaffective disorder.

^†^ Bipolar disorder/depressive disorder/anxiety disorder/obsessive compulsive disorder/stress.

After the multivariate analysis, having children aged 14 years or younger remained significantly (p = 0.001) associated, with a 71% higher risk of non-adherence to CMPA. Poor or partial adherence to psychotropic drugs tended towards a significant (p = 0.066) association with poor adherence to CMPA, conferring a 33% greater risk of non-adherence to CMPA. The number of psychiatric hospitalizations during CAPS also showed a tendency to be associated with worse adherence (p = 0.076), with a cumulative association of 5% additional non-adherence with each new hospitalization.

Occupation, type of psychiatric disorder, time of diagnosis/treatment, and FAST Interpersonal Relationships were not significant after multivariate analysis.

## Discussion

The initial hypothesis was that there would be a high percentage of CMPA non-adherence at the CAPS. The results of the study corroborate this hypothesis, since fewer than half (43%) of the participants were present at 50% or more of the meetings offered during the previous three months and 45% had never attended, even though they had clinical indications and most of the participants (72%) considered CMPA of great importance for their treatment. In one CAPS efficacy study, Tomasi et al. observed even higher absenteeism rates: more than half of the patients did not participate in any workshops (61%) or group sessions (60%). ^21^ In the present study, no association was found between time in treatment at the CAPS and CMPA adherence, unlike a previous study reporting greater group participation among those patients with longer time in treatment at a CAPS. ^21^

Although 72% of the subjects endorsed CMPA as important for their treatment, adherence was only 43%. Since the research interviewers were also staff members, the reported importance may be overestimated. Despite this bias, motivational factors should also be considered at the individual participant level. The desire to adhere, without success, may be related to cognitive deficits and the treatment approach should focus on constructing tools to improve realization of intention. On the other hand, intentional poor adherence should be addressed with interventions focused on patient awareness and psychoeducation. ^22^

Another initial study hypothesis was that more severe patients would present greater difficulty in attending CMPA. This hypothesis was partially confirmed. There was no difference between groups in terms of psychosocial functioning and symptom improvement. On the other hand, the study did find an association between number of psychiatric hospitalizations since CAPS admission and worse CMPA adherence.

There is a paradox in mental health care. Exacerbated symptoms of psychiatric illness sometimes cause patients to feel unable to attend treatments aimed precisely at the improvement of those symptoms. This seems to become worse in relation to group meetings, where people may feel less comfortable. The symptoms of mental illness may include anxiety, depression, and difficulties leaving home alone. SPMI patients are often afraid to participate in group activities as part of the symptomatology of their disease. ^12^ When exacerbated, these symptoms can compromise adherence to CMPA. Although there were no statistical differences between groups with regard to these issues, psychiatric illness symptoms were mentioned by 28% of the participants as reasons for their absence from CMPA and another 36% of the participants mentioned motivational reasons for missing these activities. Regarding mental illness, there is often confusion between lack of motivation for the task and symptoms that compromises motivation. ^22^ Given this overlap, one may assume that psychiatric symptoms, as a broad concept, were the most frequently mentioned reasons identified by patients to explain their CMPA absence. Mental health professionals should educate patients about the therapeutic benefits of coping with these fears when attending CMPA, as well as providing a secure therapeutic setting for these more severe patients.

Another pertinent study finding was the association between the number of psychiatric hospitalizations since CAPS admission and worse CMPA adherence, with a cumulative association of 5% greater non-compliance with each new hospitalization. This result is aligned with the implementation of CAPS as part of a mental health network, and its focus on promoting psychosocial rehabilitation, as well as reducing psychiatric hospitalizations. ^1 - 3^ Since this was a cross-sectional study, with a risk of reverse causality, care was taken to analyze the time of hospitalizations after admission to the CAPS and whether they coincided with the 3-month CMPA adherence evaluation period. CMPA absence was not due to patient hospitalization during the study. The findings suggest that groups and workshops can reduce hospitalizations in those patients who attend 50% or more of the meetings offered, providing analytical data indicative of CAPS’ effectiveness for prevention of psychiatric hospitalizations. A previous cohort study analyzed the effectiveness of CAPS and found reduction in crisis reports in all users, and fewer hospitalizations among those in intensive treatment regimens. ^21^ The findings suggest that the relationships established with professionals enable them to act promptly upon signs of worsening symptoms, preventing hospitalization. ^21^ Although not associated with adherence to CMPA, other studies also found low hospitalization rates after initiating treatment at CAPS. ^23 , 24^ A contrasting finding was reported by Volpe et al., ^25^ who investigated the association between community healthcare resources and risk of psychiatric readmission. These authors found that coverage of CAPS at the place of residence did not have protective effects against psychiatric readmission. They discussed access barriers and patient non-adherence as possible major reasons for this association and the present study also stresses non-adherence as a crucial factor impeding effective treatment, as revealed by the association between number of psychiatric hospitalizations after CAPS admission and lower CMPA adherence.

Adherence to psychotropic drugs was comparatively higher than CMPA adherence, at 67% and 43% respectively. Other studies indicate a predominance of drug treatments, such as use of antipsychotics, at CAPS. ^23 , 24^ The literature indicates a general drug non-adherence rate of 50% in chronic diseases. ^26 , 27^ One study estimated 65% adherence to psychotropic drugs, coinciding with the findings of this study. ^28^ On the other hand, one CAPS survey identified drug adherence of 32%, ^29^ while another only reported 12%. ^30^ Notwithstanding the diversity found in the literature on adherence to psychotropic drugs, more participants in the present study were adherent to drug treatment than to CMPA treatment.

An association was found between poor or partial adherence to psychotropic drugs and non-adherence to CMPA, conferring a 33% higher risk of non-adherence. One study found that schizophrenic patients who are adherent to medications are more likely to be adherent to psychosocial group therapy and rehabilitation. ^31^ A hypothesis that patients who do not use medications appropriately would be more symptomatic and could be less adherent to collective treatment was not supported by the study findings, since there was no association between CMPA adherence and degree of symptomatic severity. Thus, it is possible that individuals who have poor adherence to both medication and CMPA have a behavioral non-adherence profile.

Adherence to treatment is a complex behavioral process associated with multiple elements: factors related to the health system and team, patient, disease, treatment, and socioeconomic conditions. ^32^ The present study showed a strong association with having a child aged 14 years or younger and non-adherence to CMPA. Individuals with children in this age group had a 71% higher risk of non-adherence. Small children require constant care, which seems to compromise weekly adherence to CMPA. This finding also suggests a limited network of family support for these patients and also a reduced social support context. They do not seem to have family members and/or daycare/schools that can assist in caring for younger children during the CAPS treatment. Although the scope of the study precludes exploring this finding in depth, it is also noteworthy to suppose that these young children could be mainly cared for by mentally ill parents, with a possible negative impact on their psyches. ^33^ Social commitments and care for family members and young children was mentioned by 18% of the patients to explain CMPA absences. No data were found in CAPS research literature on the relationship between adherence to treatment and having children.

In clinical practice, financial difficulty is frequently identified by CAPS patients and practitioners as an impediment to adherence to CMPA. When asked about the reasons for absence, 19% of the participants reported financial issues. Although receiving permanent benefit was associated with adherence to the CMPA in the bivariate analysis, this difference was not maintained in the multivariate analysis. Most of the study participants had incomes equivalent to one minimum wage or less, and constitute a vulnerable population, with competing health, economic, and social demands. These competing demands may contribute to lower adherence to more intensive treatment such as CMPA, even among patients who receive financial benefits. Social vulnerability and its relationship with adherence to CMPA should be better addressed in future studies.

CAPS are a specific mental health care strategy in Brazil that is aimed at psychosocial rehabilitation of severe mental patients and where the main tool is CMPA. The study sought to evaluate this technology with a view to a future plan to make its operations more effective through better patient adherence. The impact of CMPA adherence seems to reduce the number of hospitalizations, which tends to favor rehabilitation and provide greater quality of life for patients and their families, with consequent reduction of societal costs. However, the low adherence to CMPA (43%) shows that the main rehabilitation tool used at CAPS is not being harnessed to its full potential. A complex structure of multidisciplinary care with significant social and financial investment appears to be being underutilized. It is crucial to identify factors that are compromising adherence to rehabilitation tools to make this structure more effective.

One of the advantages of this study is the originality of its evaluation of CAPS results using a quantitative methodology. There is little information in the literature regarding the operation of CAPS and a real gap in terms of quantitative analysis. This is an exploratory study, generating hypotheses about a topic that has not yet been explored in the literature. Assessing CMPA adherence levels and possible factors associated with adherence provides important results for future strategies to improve CAPS care.

## Limitations

This is a cross-sectional study, which identifies associations between adherence to CMPA and variables, but without the power to determine causality. In this sense, the role of this research is to generate hypotheses. However, for some variables there is only one possible direction, as in the case of having a child aged 14 years or less, which can only be a cause of non-adherence to CMPA.

One weak point of the study is the sample size, which was a little too small to analyze the high number of variables that were studied. The sample size was restricted by the size of the active population in CAPS treatment during the data collection period, the exclusion criteria, and some losses from the sample. The profile is of severe and absent patients, requiring great effort to access them. Some variables were not statistically significant, possibly due to the lack of statistical power of the study, although they indicated trends. This was the case of reports of poor social support, personality disorder, younger age, and employment or sickness benefits, all of which trended toward worse CMPA adherence. It is also possible that, with a more robust sample, other variables that were neutral in the study would have been statistically significant.

Another limitation refers to the data collection period, which is a usual period for taking vacations. There is a possibility that some patients were traveling during this period and failed to participate for that reason. However, the present study was unable to obtain this information.

Finally, all types of CMPA were considered as a single entity and differences between specific types of group activities could not be addressed. Moreover, the reasons for non-adherence to CMPA were addressed descriptively and a more comprehensive understanding of motivational patient factors is needed in future studies in order to identify the best therapeutic approach for each patient.

## Conclusion

This study stands out for its evaluation of the CPMA adherence levels at a CAPS and of factors associated with adherence. CPMA are tools used for rehabilitation of patients with SPMI. The level of CMPA adherence observed in the study was 43% and the factors associated with non-adherence were poor adherence to psychotropic drugs, number of psychiatric hospitalizations after CAPS admission, and having children aged 14 years or younger.

Patients who do not adhere well to psychotropic drugs tend to exhibit greater vulnerability in relation to their treatment as a whole. Non-adherent behavior can be mitigated by investing in the team-patient relationship, through a professional and empathetic attitude, along with patient psycho-education and motivational approaches. ^18 , 32^ It is also necessary to listen to the needs of the health professionals caring for these more vulnerable patients, in order to provide them with appropriate working conditions in terms of infrastructure and training needs. Also, awareness of patients’ motivation and experiences in group activities in order to better adjust them appears to be crucial to increasing adherence.

The association between a greater number of psychiatric hospitalizations and poor adherence to groups and workshops raises the hypothesis that collective activities may contribute to reducing hospitalizations and reinforce the central role of CAPS in mental health policy. Future research could test this hypothesis using larger samples and different designs (longitudinal, experimental) to obtain a better understanding of the effectiveness of CAPS for preventing psychiatric hospitalizations.

Finally, considering that having children aged 14 years or younger was a factor in lower adherence to the CMPA, a joint effort is needed to address this finding. At the service level, professionals should be aware of the great difficulty that these parents have to adhere to treatment, and considering the limitations that patients with SMPI face when dealing with different social situations, professionals can help them to identify solutions, including possible cooperation between the CAPS and childcare centers and provision of guidance to family members on the importance of adhering to treatment and the need to support patients. At the governmental level, investments are needed to improve the childcare network and access to these services in order to facilitate parents’ adherence to systematic health care such as CMPA.
